# Implementation of point-of-care molecular testing for respiratory viruses in congregate living settings

**DOI:** 10.1017/ice.2024.72

**Published:** 2024-09

**Authors:** Charlie Tan, Christina K. Chan, Marianna Ofner, Jaclyn O’Brien, Neethu R. Thomas, James Callahan, Brigitte Pascual, Shawn J. Palmer, Victoria Serapion, Hannah Fabro, Robert A. Kozak, Heather Candon, Adrienne K. Chan, Jeff E. Powis, Jerome A. Leis

**Affiliations:** 1 Infection Prevention and Control, Sunnybrook Health Sciences Centre, Toronto, ON, Canada; 2 Division of Infectious Diseases, Department of Medicine, University of Toronto, Toronto, ON, Canada; 3 Dalla Lana School of Public Health, University of Toronto, Toronto, ON, Canada; 4 Infection Prevention and Control, Toronto East Health Network, Toronto, ON, Canada; 5 Department of Laboratory Medicine and Pathobiology, University of Toronto, Toronto, ON, Canada; 6 Department of Public Health Sciences, Queen’s University, Kingston, ON, Canada

## Abstract

**Objective:**

To implement and evaluate a point-of-care (POC) molecular testing platform for respiratory viruses in congregate living settings (CLS).

**Design:**

Prospective quality improvement study.

**Setting:**

Seven CLS, including three nursing homes and four independent-living facilities.

**Participants:**

Residents of CLS.

**Methods:**

A POC platform for COVID-19, influenza A and B, and respiratory syncytial virus was implemented at participating CLS from December 1, 2022 to April 15, 2023. Residents with respiratory symptoms underwent paired testing, with respiratory specimens tested first with the POC platform and then delivered to an off-site laboratory for multiplex respiratory virus panel (MRVP) polymerase chain reaction (PCR) as per standard protocol. Turn-around time and diagnostic accuracy of the POC platform were compared against MRVP PCR. In an exploratory analysis, time to outbreak declaration among participating CLS was compared against a convenience sample of 19 CLS that did not use the POC platform.

**Results:**

A total of 290 specimens that underwent paired testing were included. Turn-around time to result was significantly shorter with the POC platform compared to MRVP PCR, with median difference of 36.2 hours (interquartile range 21.8–46.4 hours). The POC platform had excellent diagnostic accuracy compared to MRVP PCR, with area under the curve statistic of .96. Time to outbreak declaration was shorter in CLS that used the POC platform compared to CLS that did not.

**Conclusion:**

Rapid POC testing platforms for respiratory viruses can be implemented in CLS, with high diagnostic accuracy, expedited turn-around times, and shorter time to outbreak declaration.

## Introduction

Outbreaks of respiratory viruses are common in congregate living settings (CLS), including nursing homes and independent-living facilities. Residents of CLS were among the most heavily impacted by the COVID-19 pandemic.^1–3^ Other respiratory viruses such as influenza and respiratory syncytial virus (RSV) have also historically imposed considerable seasonal burdens on CLS residents, with over half of influenza outbreaks occurring in this sector.^4^ The incidence of co-circulating viruses returned to prepandemic levels during the 2022–2023 respiratory season, with up to 670,000 hospitalizations and 98,000 deaths due to influenza in the United States,^5^ and similar trends seen in Canada, Europe, and Australia.^4,6,7^ Heightened RSV transmission was also observed, with higher peak percentage of test positivity compared to before the COVID-19 pandemic.^8^

Outbreaks of respiratory viruses can propagate rapidly within CLS; reported attack rates for COVID-19 have been greater than 60% among at-risk residents. Timely identification of outbreaks is therefore critical to effective implementation of infection prevention and control (IPAC) measures to reduce the number of affected residents.^9–11^

In CLS, however, timely diagnostic testing for respiratory viruses after identification of a syndromic resident is a challenge, as highlighted throughout the COVID-19 pandemic.^12^ CLS typically do not have capacity for in-house laboratory testing, with specimens needing to be delivered to centralized off-site laboratories. Time to result is compounded at various steps in this cascade, including specimen delivery, manual specimen processing and preparation, batching of multiple specimens for high-throughput testing platforms, intrinsic run time of testing platforms, and result notification to the CLS.

Full-service point-of-care (POC) molecular testing platforms with rapid turn-around times implemented directly in CLS by frontline clinical staff are a potential pragmatic option to expedite detection of respiratory viruses in CLS residents. POC platforms have been validated and used in the detection of respiratory viruses, though principally in acute care hospital settings, clinics, and testing centers.^13–19^ There is a paucity of data on their applicability in the CLS sector and use by non-laboratory frontline personnel.

The objective of this study was to determine the impact of implementing a POC molecular testing platform for detection of respiratory viruses in CLS. We sought to evaluate the platform’s diagnostic accuracy, turn-around time to result, and whether time to initiation of outbreak measures could be reduced.

## Methods

### Study setting

Community-based CLS have been supported by hospital-affiliated IPAC departments, referred to as IPAC “hubs”, in Toronto, Canada since October 2020.^20^ The purpose of the IPAC hubs is to build frontline IPAC capacity and promote IPAC best practices among CLS, leveraging resources and expertise from the acute care sector. Our two IPAC hubs (henceforth referred to as Hub A and Hub B) support 30 CLS across north and east Toronto, including 14 nursing homes and 16 independent-living facilities.

### Description and implementation of intervention

We implemented a quality improvement study assessing the impact of a POC molecular testing platform in CLS from December 1, 2022 to April 15, 2023. The Cepheid GeneXpert^®^ Xpert^®^ Xpress CoV-2/Flu/RSV plus platform (Sunnyvale, CA) was implemented at three nursing homes and four independent-living facilities across Hub A and Hub B. Xpert^®^ Xpress CoV-2/Flu/RSV plus is a POC cartridge-based nucleic acid amplification test platform that detects SARS-CoV-2, influenza A, influenza B and RSV. Each cartridge is loaded onto a GeneXpert^®^ instrument and used to test a single respiratory specimen. This platform has been widely validated against conventional laboratory-based assays and has maintained accuracy in the detection of SARS-CoV-2 for both wild-type virus and subsequent circulating variants of concern.^21,22^

Each participating CLS was provided a GeneXpert^®^ IV (four module) instrument and a supply of Xpert^®^ Xpress CoV-2/Flu/RSV plus cartridges. On-site training sessions were organized at each CLS to support implementation of the POC testing platform. All subsequent specimen collection and preparation, testing of specimens on the platform, and result interpretation were performed by frontline CLS clinical staff without preexisting laboratory experience. IPAC practitioners from the hubs performed maintenance of the GeneXpert^®^ instruments and reviewed the results for quality assurance.

Residents with respiratory symptoms underwent paired testing, whereby nasopharyngeal (NP) or mid-turbinate (MT) swabs were tested first with Xpert^®^ Xpress CoV-2/Flu/RSV plus, then delivered to an external off-site laboratory for testing as per standard protocol. Testing was ordered at the discretion of nursing staff or physicians/nurse practitioners and was performed by nursing staff. Specimens could be collected at any time during the day, though were only delivered to external laboratories during daytime working hours. Laboratory-based testing was performed at a hospital-affiliated laboratory or reference provincial laboratory. A multiplex respiratory virus panel (MRVP) real-time polymerase chain reaction (PCR) was performed, which detected 16 viral targets: influenza A, influenza A H3 subtype, influenza A H1 (pdm09) subtype, influenza B, RSV A/B, parainfluenza (1–4), adenovirus, enterovirus, seasonal coronavirus (OC43, 229E, NL63, HKU1), rhinovirus, and human metapneumovirus. Both laboratories use independent proprietary assays that have been internally validated.^23^ CLS were notified regarding MRVP PCR results via either electronic communication from their corresponding IPAC hub or fax from the testing laboratory.

### Data collection

For each NP/MT specimen collected, we documented CLS of collection, date and time loaded onto the POC testing platform, date and time of POC result, specific virus detected on POC platform if positive, date and time of MRVP PCR result, and specific virus detected on MRVP PCR if positive. In addition, we collected date and time of declaration and date and time of outbreak-definition-meeting MRVP PCR result (if available) for all outbreaks of COVID-19, influenza A, influenza B, and RSV that occurred during the study period. We collected this same data for a convenience sample of eight CLS in Hub A and 11 CLS in Hub B that did not use the POC platform. Outbreaks were declared by the local public health unit based on regional outbreak definitions for respiratory viruses.^10,24–26^ Specifically, for COVID-19, outbreaks were declared with identification of two or more epidemiologically linked residents with test-confirmed COVID-19 infection within a 7-day period.^24^ For other respiratory viruses, outbreaks were declared with identification of two or more epidemiologically-linked residents with acute respiratory infection, at least one of which must be test-confirmed, within a 48-hour period.^25^ Outbreaks could be declared based on results from PCR, POC platform, or rapid antigen test (RAT) for COVID-19.

### Data analysis

Baseline characteristics of the participating CLS were described. Medians and interquartile ranges (IQR) were used for continuous variables, while frequencies and percentages were used for categorical variables. For each NP/MT specimen collected, we compared turn-around time to result between the POC platform and reference laboratory MRVP PCR. Time of specimen being loaded onto the POC platform was taken as the starting time. For POC testing, time of result output from the platform was taken as the ending time. For MRVP PCR, time of result notification via electronic communication from the IPAC hub or fax from the testing laboratory was taken as the ending time. Turn-around times were described using medians and IQRs, and differences between the two platforms were evaluated using the Wilcoxon signed-rank test for paired comparisons. We also calculated sensitivity, specificity, positive predictive value (PPV), negative predictive value (NPV), and area under the curve (AUC) statistic of the POC platform, using MRVP PCR as the reference standard. We conducted a sensitivity analysis using a cycle threshold of less than 36 instead of the platform’s interpretation to adjudicate a positive result for SARS-CoV-2 on the POC platform. Lastly, as an exploratory analysis, we compared median time to outbreak declaration from outbreak-definition-meeting MRVP PCR result (calculated as time outbreak was declared by local public health unit subtracted by time qualifying MRVP PCR result became available) between CLS that did use the POC platform to a convenience sample of CLS that did not use the platform. Threshold for statistical significance was set at *P* < .05. All resident-identifying information was anonymized.

All statistical analyses were performed using Stata 18 (College Station, TX). Research ethics review was not required because the study met criteria for exemption as improvement in quality and not human subject research.

## Results

The characteristics of the included CLS are shown in Table 1. The POC testing platform was implemented at seven CLS, including three nursing homes and four independent-living facilities. In total, there were 937 combined beds across included CLS.

**Table 1.** Baseline characteristics of congregate living settings in Hub A and Hub B that used POC platform

Characteristic	Hub A (n = 4)	Hub B (n = 3)	Total (n = 7)
Type of CLS, number (%)			
Nursing home	1 (25.0)	2 (66.7)	3 (42.9)
Independent living facility	3 (75.0)	1 (33.3)	4 (57.1)
Number of beds per CLS, median (IQR)	139 (104–157)	158 (119–181)	156 (101–159)
Number of CLS with designated IPAC Lead, number (%)	1 (25.0)	2 (66.7)	4 (57.1)
Number of staff trained to use POC platform, median (IQR)	4 (3–4)	3 (3–3)	3 (3–3.5)
Number of IPAC hub practitioners	4	5	9

Abbreviations: CLS, congregate living setting; IPAC, infection prevention and control; IQR, interquartile range; POC, point-of-care.

A total of 291 consecutive NP/MT specimens underwent paired testing with the POC platform and MRVP PCR. One specimen was excluded due to indeterminate MRVP PCR result, leaving 290 specimens for analysis. On the POC platform, 107 (36.9%) specimens were positive. SARS-CoV-2 was the most common virus identified (88.8%, 95/107), followed by RSV (6.5%, 7/107) and influenza A (4.7%, 5/107). There were no specimens positive for influenza B. Respiratory viruses other than SARS-CoV-2, influenza, and RSV that were detected on MRVP PCR included seasonal coronavirus (6.2%, 18/290), rhinovirus/enterovirus (2.8%, 8/290), parainfluenza (0.7%, 2/290), and human metapneumovirus (0.3%, 1/290).

Turn-around time to result with the POC platform and MRVP PCR is shown in Table 2. Median turn-around time with the POC platform was 36 minutes (IQR 36–36 minutes). In contrast, median turn-around time with MRVP PCR was 36.8 hours (IQR 22.4–47.0 hours) (*P* < .001). Median difference in turn-around time was 36.2 hours (IQR 21.8–46.4 hours).

**Table 2.** Turn-around time to result with POC platform and MRVP PCR

Platform	Median time (IQR)	*P*-value
POC platform	36 minutes (36–36 minutes)	*P* < .001
MRVP PCR	36.8 hours (22.4–47.0 hours)	
Difference in turn-around time	36.2 hours (21.8–46.4 hours)	NA

Abbreviations: IQR, interquartile range; MRVP, multiplex respiratory virus panel; NA, not applicable; POC, point-of-care; PCR, polymerase chain reaction.

The sensitivity, specificity, PPV, NPV, and AUC of the POC platform compared to MRVP PCR as the reference standard are shown in Table 3. The POC platform demonstrated excellent diagnostic accuracy. AUC statistic was 0.96 (95% CI, .94–.98), indicating robust discrimination. Sensitivity analysis using a cycle threshold cut-off of less than 36 to adjudicate SARS-CoV-2 positivity yielded similar results.

**Table 3.** Diagnostic accuracy of POC platform compared to MRVP PCR as reference standard

	AUC (95% CI)	Sensitivity % (95% CI)	Specificity % (95% CI)	PPV % (95% CI)	NPV % (95% CI)
Overall	0.96 (0.94–0.98)	98.9 (97.7–100.0)	92.4 (89.3–95.4)	86.0 (82.0–90.0)	99.5 (98.6–100.0)
Cycle threshold <36 for SARS-CoV-2	0.97 (0.95–0.99)	98.9 (97.7–100.0)	94.8 (92.2–97.0)	89.7 (86.1–93.0)	99.5 (98.6–100.0)

Abbreviations: AUC, area under the curve; CI, confidence interval; MRVP, multiplex respiratory virus panel; NPV, negative predictive value; PCR, polymerase chain reaction; POC, point-of-care; PPV, positive predictive value.

During the study period, there were 11 outbreaks among CLS in Hub A and Hub B that used the POC platform, all of which were COVID-19. Seven (63.6%) were declared before the qualifying MRVP PCR result. Of the remaining four outbreaks, time to outbreak declaration was 6.6 hours (IQR 4.6–9.2 hours). In comparison, there were 24 respiratory virus outbreaks across 16 CLS that did not use the POC platform, of which 20 were COVID-19, two were RSV, one was influenza A, and one was mixed influenza A/RSV. Of these, five outbreaks (20.0%) were declared before the qualifying MRVP result, based on RATs (for COVID-19) or clusters of syndromic patients. Of the remaining 19 outbreaks, median time to outbreak declaration from MRVP result was 19.1 hours (IQR 11.7–43.3 hours).

## Discussion

In this multicenter study, a rapid POC molecular testing platform for respiratory viruses in CLS was successfully implemented across seven CLS comprising 937 combined resident beds, resulting in respiratory virus identification more than 24 hours earlier than conventional testing at an off-site laboratory. Molecular POC testing revealed excellent diagnostic accuracy compared to reference laboratory testing and was associated with earlier identification of respiratory outbreaks.

To our knowledge, our study is the first to evaluate rapid POC molecular testing platforms for detection of respiratory viruses in CLS. Previous studies in acute care facilities similarly found improvements in turn-around times and high diagnostic accuracy.^27–29^ In our participating CLS, frontline clinical staff without existing laboratory experience were responsible for all stages of the testing process, including specimen collection and preparation, use of the POC platform, and interpretation of results. Since most CLSs do not have laboratory capacity and need to send specimens to external laboratories, our findings demonstrate the suitability and feasibility of on-site POC testing platforms in the CLS sector.

Our study found rapid time to result of only 36 minutes with the POC platform, compared to over 35 hours with conventional laboratory testing. Rapid detection of respiratory viruses is critical for CLS, with delays to implementation of control measures associated with heightened risk of transmission, increased frequency and size of outbreaks, and greater mortality.^30–32^ Improving turn-around time to less than 24 hours has been identified as a key priority for CLS during the COVID-19 pandemic.^33^

Despite this evidence, such timely diagnosis remains a challenge for CLS. In a survey of nursing homes in the United States during the early COVID-19 pandemic, the majority of facilities had greater than 24-hour turn-around time to result, with approximately 40% having turn-around times of three days or longer.^12^ Processes for centralized off-site laboratory testing confer inherent delays that lead to increased time to result for CLS. Even in our setting, where CLS have ready access to multiple external laboratories and a provincially-funded courier service for specimen delivery is available, turn-around time through laboratory testing was still greater than 24 hours.

We found that time to outbreak declaration was shorter in CLS that used the POC testing platform; seven of 11 outbreaks were declared before qualifying MRVP result, and the remaining outbreaks were declared earlier than comparator homes that did not use the platform. Studies of POC testing platforms in acute care settings have demonstrated enhanced implementation of measures associated with improved patient outcomes and control of disease transmission.^27,34,35^

There were several limitations to this study. This was a non-randomized study and CLS that implemented the POC testing platform did so on a voluntary basis, potentially introducing facility-level volunteer bias. The study period of December 1, 2022 to April 30, 2023 may have missed respiratory virus activity during the early respiratory season. The turn-around time for the POC platform was likely underestimated, as it did not include time for specimen collection and preparation, as well as time from result output to being reviewed by staff. However, an increase of only a few minutes would be anticipated as the platform was used in real time. Time for specimen collection also impacts both the POC platform and MRVP PCR, and therefore would not change the difference in turn-around time found. Another limitation is that the comparison of time to outbreak declaration was an uncontrolled exploratory analysis against a convenience sample of CLS that did not use the POC platform. This could be affected by facility- and resident-level differences between individual CLS. Furthermore, we calculated time to outbreak definition from outbreak-definition-meeting MRVP PCR result, as we did not have resident-level information on time of symptom onset. Additional research evaluating the impact of POC testing platforms on outbreak size, implementation of outbreak control measures, such as administration of antiviral chemoprophylaxis for influenza, and resident-level outcomes, such as secondary attack rate, hospitalization, and death is needed. The findings in our study apply primarily to COVID-19, which was the predominant virus during the study period, and the POC platform was unable to detect viruses other than SARS-CoV-2, influenza, or RSV. Lastly, our study was only conducted over one respiratory season; sustained feasibility in CLS given perpetual financial and human resources limitations is another area for future study.

Rapid POC molecular testing platforms for detection of respiratory viruses can be implemented in CLS, with excellent diagnostic accuracy, expedited turn-around times, and shorter time to outbreak declaration. This approach would be scalable across the CLS sector, although larger system-wide evaluations of POC testing platforms are needed.
